# Blunted endogenous opioid release following an oral dexamphetamine challenge in abstinent alcohol-dependent individuals

**DOI:** 10.1038/s41380-018-0107-4

**Published:** 2018-06-25

**Authors:** Samuel Turton, James FM Myers, Inge Mick, Alessandro Colasanti, Ashwin Venkataraman, Claire Durant, Adam Waldman, Alan Brailsford, Mark C Parkin, Gemma Dawe, Eugenii A Rabiner, Roger N Gunn, Stafford L Lightman, David J Nutt, Anne Lingford-Hughes

**Affiliations:** 1 Neuropsychopharmacology Unit, Centre for Psychiatry, Imperial College London, London, UK; 2 Institute for Clinical Teratology and Drug Risk Assessment in Pregnancy, Charité Universitätsmedizin, Berlin, Germany; 3 Department of Neuroscience, Brighton and Sussex Medical School, University of Sussex, Brighton, UK; 4 Centre for Affective Disorders, Institute of Psychiatry, Psychology and Neuroscience, King’s College London, London, UK; 5 Centre for Clinical Brain Sciences, University of Edinburgh, Edinburgh, UK; 6 Analytical and Environmental Sciences, King’s College London, London, UK; 7 Department of Neuroradiology, Imperial College Healthcare NHS Trust, London, UK; 8 Imanova Limited, London, UK; 9 Centre for Neuroimaging Sciences, King’s College London, London, UK; 10 Centre for Restorative Neuroscience, Division of Brain Sciences, Imperial College London, London, UK; 11 Henry Wellcome Laboratories for Integrative Neuroscience & Endocrinology, University of Bristol, Bristol, UK

**Keywords:** Addiction, Neuroscience

## Abstract

Addiction has been proposed as a ‘reward deficient’ state, which is compensated for with substance use. There is growing evidence of dysregulation in the opioid system, which plays a key role in reward, underpinning addiction. Low levels of endogenous opioids are implicated in vulnerability for developing alcohol dependence (AD) and high mu-opioid receptor (MOR) availability in early abstinence is associated with greater craving. This high MOR availability is proposed to be the target of opioid antagonist medication to prevent relapse. However, changes in endogenous opioid tone in AD are poorly characterised and are important to understand as opioid antagonists do not help everyone with AD. We used [^11^C]carfentanil, a selective MOR agonist positron emission tomography (PET) radioligand, to investigate endogenous opioid tone in AD for the first time. We recruited 13 abstinent male AD and 15 control participants who underwent two [^11^C]carfentanil PET scans, one before and one 3 h following a 0.5 mg/kg oral dose of dexamphetamine to measure baseline MOR availability and endogenous opioid release. We found significantly blunted dexamphetamine-induced opioid release in 5 out of 10 regions-of-interest including insula, frontal lobe and putamen in AD compared with controls, but no significantly higher MOR availability AD participants compared with HC in any region. This study is comparable to our previous results of blunted dexamphetamine-induced opioid release in gambling disorder, suggesting that this dysregulation in opioid tone is common to both behavioural and substance addictions.

## Introduction

Alcohol dependence (AD) affects 4% of adults in Europe and 4.7% in the United States, and globally 3.3 million deaths per year (5.9% of deaths worldwide) are attributed to harmful alcohol use [1]. Treatment for alcohol abuse and dependence costs an estimated $12 billion in the US and €5 billion in the EU [2, 3], however three quarters of individuals with AD will not remain abstinent from alcohol in the first year following treatment [4]. Thus, there is a substantial unmet need in reducing this harm.

Psychosocial approaches are the mainstay treatment of AD but effective relapse prevention medications, including the opioid antagonists naltrexone and nalmefene, are available [5–7]. Opioid antagonists modulate the mesolimbic ‘reward’ pathway, which is proposed to underpin their effectiveness in reducing the risk of relapse to heavy drinking [5, 7–9]. Using functional magnetic resonance imaging (fMRI), naltrexone and nalmefene have been shown to reduce brain responses in the mesolimbic pathway to salient alcohol cues or exposure [10, 11]. These medications do not help everyone and so a better understanding of opioid system function in alcoholism is required to develop improved therapies and better target individuals with current medication.

The mu-opioid receptor (MOR) subtype is expressed in brain regions associated with addiction including the ventral tegmental area, nucleus accumbens and amygdala. The MOR and its endogenous ligands, including β-endorphin, play an important role in reward [12–14]. Some substances of abuse, including alcohol and amphetamines, increase the levels of endogenous opioids binding to MORs and this is associated with positive subjective effects, including ‘best ever’ feelings and euphoria [15, 16]. Naltrexone has been shown to attenuate these positive subjective effects [17–19]. Endogenous opioid dysregulation may also play an important role in the vulnerability to developing addiction where a ‘reward deficient’ state leads to substance abuse to compensate for opioidergic hypofunction [20, 21].

Lower basal endogenous opioid levels in the brain may result in higher opioid receptor availability and this has been demonstrated in AD during early abstinence using the non-selective radioligand [^11^C]diprenorphine [22] and the MOR-selective radioligand [^11^C]carfentanil [23, 24]. Higher opioid receptor availability is associated with alcohol craving [22, 23, 25] and alcohol-dependent individuals with higher [^11^C]carfentanil binding may benefit more from naltrexone treatment [25]. This suggests that the effectiveness of opioid antagonists is linked to opioid receptor availability in humans. However, no studies have assessed in vivo endogenous opioid tone in AD, which is also key to understanding the predictors of response to opioid antagonists.

We have developed and validated a [^11^C]carfentanil positron emission tomography (PET) protocol to assess endogenous opioid release following an oral dexamphetamine challenge [15, 26]. With this protocol, we demonstrated no difference in baseline MOR availability but a blunted dexamphetamine-induced opioid release, with blunted associated subjective effects, in gambling disorder [27]. In this study, we applied the same protocol to test the hypotheses that in AD there is blunted dexamphetamine-induced endogenous opioid release, as shown in gambling disorder, and a higher baseline MOR availability.

## Patients and methods

This study was approved by the West London Research Ethics Committee and the Administration of Radioactive Substances Advisory Committee, UK (14/LO/1552). Written informed consent was obtained from all the participants.

Alcohol-dependent (AD) men (*n* = 13, > 4 weeks abstinent, DSM-5 criteria for ‘severe’ alcohol use disorder) were recruited from Central North West London NHS Foundation Trust, UK and associated services. Severity of AD, relapse risk and alcohol craving were assessed using the Severity of Alcohol Dependence Questionnaire (SADQ) [28], Time to Relapse Questionnaire (TRQ) [29] and Alcohol Urge Questionnaire (AUQ) [30]. ‘High risk’ alcohol exposure was calculated as lifetime cumulative weeks with > 60 g average daily alcohol consumption [31]. Male healthy controls (HC) (*n* = 15) included 10 from previous studies [15, 26] and 5 recruited for the current study to achieve age-matching between groups.

All participants’ physical and mental health history, including history of alcohol, tobacco and substance use, was assessed by psychiatrists using the Mini International Neuropsychiatric Interview (MINI-5) [32]. Current or past history of gambling disorder or substance dependence (excluding nicotine) was an exclusion criterion; previous recreational drug use was allowed ( > 10 times in lifetime: cannabis: 3 HC, 9 AD, cocaine: 6 AD, stimulants: 5 AD, inhalants: 1 HC, 3 AD, hallucinogens: 1 AD, sedatives: 1 AD, 2 HC, opioids: 1 AD). HC were excluded if they drank > 21 UK units of alcohol (166 g) per week or had a previous history of AD. Drug use (except nicotine) was not permitted 2 weeks prior to the study visits and confirmed by negative urine drug screen (cocaine, amphetamine, THC, methadone, opioids, benzodiazepines). Participants were breathalysed for alcohol. Smoking tobacco was not allowed 1 h before each scan. All participants had laboratory and ECG results within normal range, and were not prescribed any regular psychotropic medications.

Participants with current or previous psychiatric disorders were excluded. However, in AD participants a past history of depression (non-psychotic) and/or anxiety disorders was permitted owing to the high prevalence in this population. Depression was assessed with the Beck Depression Inventory (BDI) [33] and anxiety with Spielberger State/Trait Inventory (SSAI and STAI) [34]. Impulsivity was assessed with the UPPS-P Impulsive Behaviour Scale [35].

### Genotyping

Blood samples for genotyping of OPRM1 A118G polymorphism were available from all participants (AD: *n* = 13; HC: *n* = 11) except for four HC from our first study [15] and were analysed by LGC Limited (Middlesex, UK). DNA was extracted and normalised and underwent SNP-specific KASP^TM^ Assay mix. Loci with a call rate < 90% were not included. Participants were categorised as a G-allele carrier (G:A or G:G) or not (A:A).

### PET and MR imaging protocol

We followed our protocol as previously described [15, 26, 27]. [^11^C]carfentanil PET scans were acquired on a HiRez Biograph six PET/CT scanner (Siemens Healthcare, Erlangen, Germany). Dynamic emission data were collected continuously for 90 min (26 frames, 8 × 15 s, 3 × 60 s, 5 × 120 s, 5 × 300 s, 5 × 600 s) following an intravenous bolus infusion of maximum 350MBq [^11^C]carfentanil infused over 20 sec. Participants underwent two [^11^C]carfentanil PET scans, one before and one 3 h following the oral administration of 0.5 mg/kg dexamphetamine. Seven participants (*n* = 1 AD, *n* = 7 HV) underwent scans on separate days. However, there were no significant effects of this on [^11^C]carfentanil binding potential (BP_ND_).

Subjective responses to dexamphetamine challenge were measured with the Simplified Amphetamine Interview Rating Scale (SAIRS) (15 mins pre-dose (baseline), 1, 2, 3, 4.5 h post dose) [36], and SSAI (before and after each PET scan).

Blood samples to measure plasma dexamphetamine levels were obtained pre dose (baseline), 1, 2, 3 and 4.5 h post dosing. Dexamphetamine samples were analysed at the Drug Control Centre, Analytical and Environmental Sciences, King’s College London, UK. Serum cortisol samples were collected immediately pre-dexamphetamine dose (baseline), 30, 60, 90, 120, 150 and 180 min post dose. Cortisol samples were analysed using the ARCHITECT cortisol assay at the Pathology Department, Hammersmith Hospital, Imperial College Healthcare NHS Trust, London, UK. See Supplementary data for further details of cortisol and amphetamine assays.

On a different day to their PET scans, participants underwent a T1-weighted structural MRI (Magnetom Trio Syngo MR B13 Siemens 3 T; Siemens AG, Medical Solutions). Subjects completed the ICCAM fMRI imaging platform [37, 38] and these results will be reported elsewhere.

### Image analysis

As described previously [15, 26, 27], image pre-processing and PET modelling were carried out using MIAKAT (www.miakat.org). Dynamic PET data underwent motion correction and rigid-body coregistration to the structural MRI. Ten bilateral regions of interest were chosen a priori: caudate, putamen, thalamus, cerebellum grey matter, frontal lobe grey matter, nucleus accumbens, anterior cingulate, amygdala, insular cortex and hypothalamus. All time-activity data, except the hypothalamus, were sampled using a neuroanatomical atlas [39]. This was applied to the PET image by non-linear deformation parameters derived using unified segmentation (SPM-12) of the structural MRI. The template and atlas fits were confirmed visually for each participant. The hypothalamus was manually defined on individual structural MRIs as previously described [15, 39].

[^11^C]carfentanil BP_ND_ values were quantified using the simplified reference tissue model with occipital lobe as the reference region [15, 40]. BP_ND_ is the ratio of specifically bound radioligand (e.g., bound to MOR) to that of non-displaceable radioligand (e.g., unbound and non-specifically or non-MOR bound [^11^C]carfentanil) in tissue at equilibrium. BP_ND_ used in reference tissue methods compares the concentration of radioligand in receptor-rich with receptor-free regions [41]. Reductions in [^11^C]carfentanil BP_ND_ observed following dexamphetamine challenge are owing to reductions in specific [^11^C]carfentanil binding to MORs associated with endogenous opioid release, compared with non-specific [^11^C]carfentanil binding in the occipital lobe reference region. Endogenous opioid release was indexed as the fractional reduction in [^11^C]carfentanil BP_ND_ following dexamphetamine:

$$\hskip 40pt\Delta BP_{ND}\,=\,\frac{{\left( {BP_{NDpost} - BP_{NDpre}} \right)}}{{BP_{NDpre}}}$$

### Statistical analysis

All statistical analyses were carried out using IBM SPSS (version 24). Data were normally distributed (Shapiro–Wilk) except for BDI and abstinence duration.

Demographic differences between groups were assessed using independent sample *t* tests. Omnibus mixed-model analysis of variance (ANOVA) tested the effects of status (AD or HC) on [^11^C]carfentanil BP_ND_ and ∆BP_ND_, SAIRS and SSAI subjective responses, plasma amphetamine and serum cortisol concentrations. Post hoc tests were made using one-way ANOVA or *t* tests. Correlational analyses were made using Pearson correlation coefficient or Spearman’s rho (BDI and abstinence duration). Bonferroni corrected *p* values are reported in the results. Data were tested for sphericity, and where sphericity was violated ANOVAs were Greenhouse–Geisser corrected.

## Results

### Demographic and clinical variables

Demographic results are presented in Table 1. There were no significant differences in age or IQ between HC and AD participants. More AD participants smoked tobacco but there were no differences between daily numbers of cigarettes or dependence scores (FTND) between HC and AD smokers. AD participants had significantly higher BDI and STAI scores than HC but none reached a threshold for clinical anxiety or depression. UPPS-P negative urgency scores were higher in AD participants compared with HC.

**Table 1:** Demographic and genotype data (mean ± SD)

	Healthy controls	Alcohol dependence	*p* value (two-tailed)
Numbers	15	13	
Age	42.8 ( ± 10.2)	46.6 ( ± 7.3)	0.281
IQ	115.6 ( ± 9.9)	107.8 ( + 10.7)	0.090
Alcohol UK units/week	6.67 ( ± 8.2)	0	
Current smokers	3	7	
Cigarettes per day (current smokers)	10.0 ( ± 5.0)	10.6 ( ± 7.7)	0.247
Pack years (current and ex-smokers)	7.9 ( ± 8.1)	23.6 ( ± 14.5)	0.063
FTND (current smokers)	3.0 ( ± 2.7)	3.7 ( ± 3.1)	0.906
BDI on PET visit	0.2 ( ± 0.6)	3.3 ( ± 3.6)	**0.004**
STAI	30.3 ( ± 7.4)	37.2 ( ± 5.9)	**0.012**
SSAI (before PET 1)	27.9 ( ± 6.6)	28.8 ( ± 9.9)	0.797
UPPS-negative urgency	20.8 ( ± 6.1)	27.4 ( ± 4.1)	**0.006**
SADQ		38.5 ( ± 11.2)	
TRQ		18.9 ( ± 5.1)	
Alcohol abstinence (days)	9.6 ( ± 12.7)	604.6 ( ± 866.5)	**<** **0.001**
OPRM1 G-allele carrier	2 of 11 (18.2%)	4 of 13 (30.1%)	

Abbreviations: *FTND* Fagerstrom Test for Nicotine Dependence, *BDI* Beck Depression Inventory, *SSAI*, *STAI* Spielberger State and Trait Anxiety Inventory, *UPPS* Impulsivity Scale, *SADQ* Severity of Alcohol Dependence Questionnaire, *TRQ* Time to Relapse Questionnaire

### Injected mass and radioactivity and head motion

There were no significant differences in injected cold carfentanil mass between AD participants and HC for either scan, but there was a significantly higher injected activity in post-dexamphetamine scans in AD participants (Supplementary Table 1). There were no significant correlations between [^11^C]carfentanil BP_ND_ in any region and injected mass or activity in the pre- or post-dexamphetamine scans. There was no significant effect of dexamphetamine on movement within scans, or differences between AD and HC participants (Supplementary Tables 2 and 3).

### Dexamphetamine and cortisol pharmacokinetics

A repeated measures ANOVA showed a significant effect of Time on dexamphetamine plasma concentrations but no significant effect of Status (AD or HC) (Supplementary Figure 4, Supplementary Table 5).

Serum cortisol concentration increased following oral dexamphetamine administration (Supplementary Figure 6). A mixed-model ANOVA showed a significant effect of Time on cortisol levels but no significant effect of Status (AD or HC) (Supplementary Table 5).

### Subjective effects of amphetamine

**Fig. 1:**
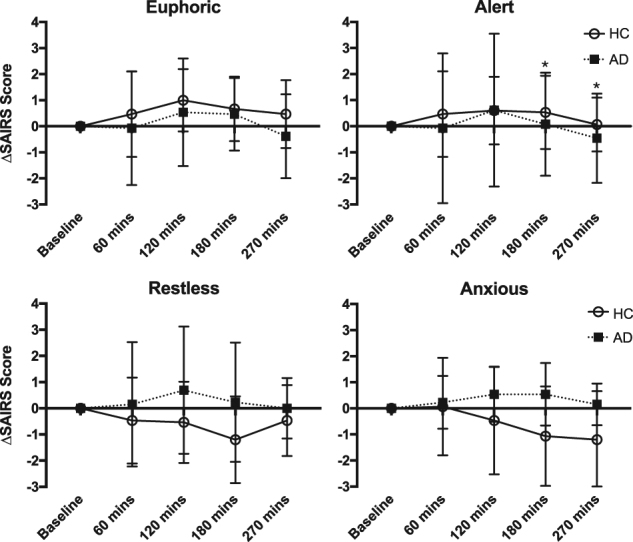
Change in SAIRS following dexamphetamine administration (mean ± SD, *p < 0.05 in HC)

The subjective effects from the oral dexamphetamine were mild in both groups (Fig. 1). A mixed-model ANOVA showed no significant effects of Time or Status (AD or HC) on changes in SAIRS scores from baseline (Supplementary Table 7). There was a significant Time × Status interaction on change in SAIRS Anxiety from baseline. Post hoc analysis (paired *t* test) showed reductions in anxiety ratings in HC at 180 and 270 mins post-dexamphetamine (*p* = 0.048 and *p* = 0.025, respectively, Bonferroni corrected *p* < 0.01), which was not present in the AD group. There was no significant effect of Time or Status on SSAI scores.

### Pre- and post-dexamphetamine [^11^C]carfentanil BP_ND_

A mixed-model ANOVA examining pre- and post-dexamphetamine [^11^C]carfentanil BP_ND_ showed a significant effect of Scan (pre- or post-dexamphetamine) demonstrating differences in BP_ND_ following dexamphetamine, and a significant Scan × Status interaction indicating a significantly different change in BP_ND_ after dexamphetamine between AD and HC (Supplementary Table 8). Post hoc paired *t* tests showed significant reductions in BP_ND_ after oral dexamphetamine in HC across all ROIs except hypothalamus and amygdala, but no significant reductions in AD participants (Supplementary Table 9).

**Fig. 2:**
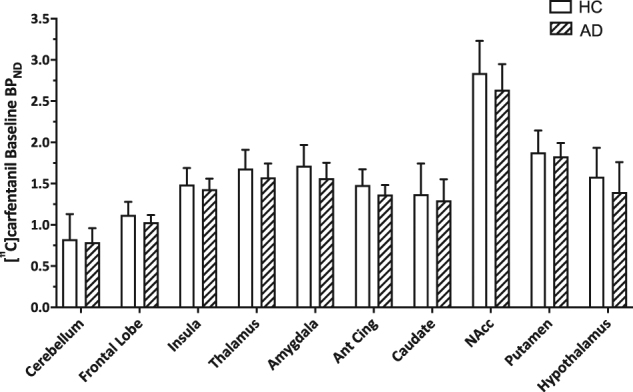
Baseline [^11^C]carfentanil BP_ND_ in HC and AD participants (mean ± SD)

A mixed-model ANOVA examining pre-dexamphetamine [^11^C]carfentanil BP_ND_ showed no significant effect of Status indicating no differences between AD and HC participants (Fig. 2, Supplementary Tables 8 and 9).

**Fig. 3:**
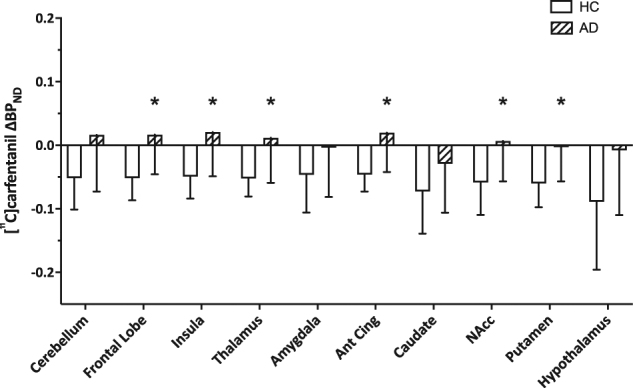
[^11^C]carfentanil ∆BP_ND_ in HC and AD participants (mean ± SD, *significant ∆BP_ND_ differences between HC and AD, Bonferroni corrected *p* < 0.005)

**Fig. 4:**
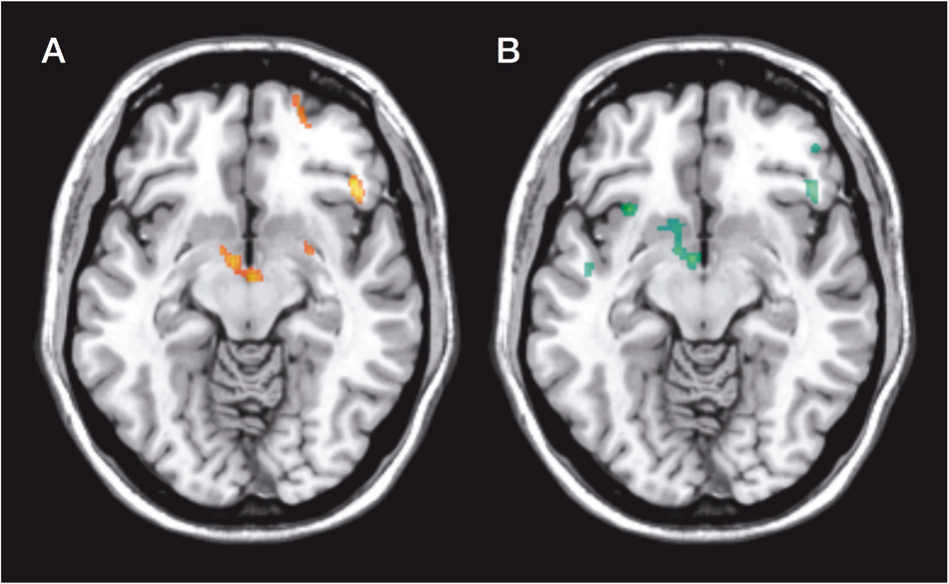
**a** Clusters with significant reductions in [^11^C]carfentanil BP_ND_ following dexamphetamine in HC. There are no significant clusters in AD. **b** Clusters with significantly lower [^11^C]carfentanil ∆BP_ND_ in AD compared with HC (all images: min cluster size 100, *p* < 0.001, z = 62)

A mixed-model ANOVA examining [^11^C]carfentanil ∆BP_ND_ showed a significant effect of Status, indicating differences between AD and HC participants. Independent sample *t* tests showed [^11^C]carfentanil ∆BP_ND_ (i.e., opioid release) was significantly blunted in AD participants, compared with HC, in the frontal lobe, insula, thalamus, anterior cingulate and putamen (Bonferroni corrected *p* < 0.005) with large effect sizes (Cohen’s D > 0.8, significant *t* tests comparing [^11^C]carfentanil ∆BP_ND_ between HC and AD) (Figs. 3 and 4, Supplementary Tables 8 and 9).

### [^11^C]carfentanil binding, demographic and clinical variables

We found no significant correlations between [^11^C]carfentanil BP_ND_ and ∆BP_ND_ with SADQ, TRQ, duration/length of alcohol abstinence or exposure measures in AD participants. Of note, AD did not report any AUQ craving at baseline or at any other point during the study. There were no significant differences in [^11^C]carfentanil BP_ND_ or ∆BP_ND_ comparing smokers with non-smokers regardless of group (combined AD and HC or AD and HC separately). The inclusion of smoking status in the mixed-model ANOVA examining [^11^C]carfentanil ∆BP_ND_ did not impact on these results (Supplementary Table 8). There were no significant correlations between [^11^C]carfentanil BP_ND_ or ∆BP_ND_ with daily cigarettes smoked or FTND scores in all participants (AD and HC) or AD or HC separately.

There was no significant association between UPPS-P negative urgency and baseline [^11^C]carfentanil BP_ND_ in AD or HC participants. There was a significant positive correlation between [^11^C]carfentanil ∆BP_ND_ in the amygdala and BDI score (Spearman’s rho = 0.654, *p* = 0.015) in AD only, indicating a higher BDI score is associated with lower opioid release. This result did not survive Bonferroni correction (two-tailed *p* < 0.005). There were no correlations between STAI and [^11^C]carfentanil BP_ND_ or ∆BP_ND_ in either participant group.

### MOR polymorphism

There was a higher G-allele prevalence in AD participants compared with HC (30.1 and 18.2% respectively—,Table 1). To assess the influence of the OPRM1 A118G polymorphism on [^11^C]carfentanil BP_ND_ the two mixed-model ANOVAs examining [^11^C]carfentanil BP_ND_ and ∆BP_ND_ were repeated with the addition of the genotype (A:A or A:G/G:G) as a between-subject factor.

We found a significant main effect of Genotype on baseline [^11^C]carfentanil BP_ND_, but no Status × Genotype interaction. A post hoc analysis independent sample *t* test showed significantly lower [^11^C]carfentanil BP_ND_ in G-allele carriers in the thalamus (two-tailed Bonferroni corrected *p* < 0.005), and a trend toward lower [^11^C]carfentanil BP_ND_ across all other regions (Supplementary Figure 10, Tables 11 and 12). We found no significant main effect of Genotype or Status x Genotype interaction on [^11^C]carfentanil ∆BP_ND_.

## Discussion

We have demonstrated a blunted dexamphetamine-induced endogenous opioid release in the brain for the first time in abstinent alcohol-dependent individuals with [^11^C]carfentanil PET. We did not find higher baseline MOR availability in AD as previously reported by ourselves and others [22, 23, 42]. Our data in AD are consistent with our previous study in gambling disorder where we also found blunted dexamphetamine-induced opioid release [27]. This strongly suggests that dysregulation of opioid tone underpins both behavioural and substance addictions.

This ‘opioid deficient’ state may play a key role in a broader ‘reward deficient’ state associated with the development and maintenance of AD and other addictions, and may precede the development of addiction [20, 21, 27, 43–45]. Individuals with AD or a family history of AD both have blunted responses to financial rewards and lower peripheral plasma β-endorphin concentrations [43, 44, 46]. Although peripheral and central β-endorphin are not directly comparable, both are produced from the cleavage of pro-opiomelanocortin (POMC) and the release of both can be stimulated by intense exercise, dexamphetamine and alcohol [15, 16, 46–49]. This low opioidergic function may represent a vulnerability to the development of addiction, which endures after successful treatment and may predispose an individual to relapse. There may be an additional impact of addiction or heavy alcohol use on endogenous opioid tone, though this is difficult to determine from our current [^11^C]carfentanil PET studies.

There is evidence of blunted intravenous dexamphetamine-induced dopamine release in AD during early abstinence [50], which may be a mechanism for the blunted dexamphetamine-induced endogenous opioid release AD. Another study reported that lower dexamphetamine-induced ventral striatal dopamine release was associated with blunted cortisol release [51]. However, in our alcohol-dependent participants, dexamphetamine-induced cortisol release was not blunted compared with controls. This may suggest that dopamine responses to dexamphetamine are less blunted in our alcohol-dependent participants, with longer durations of abstinence, than those described in early abstinence [50].

The blunted dexamphetamine-induced endogenous opioid release in AD may be mediated by a mechanism downstream from dopamine release, which would be consistent with the findings of blunted endogenous opioid release in gambling disorder despite a higher dopamine response to oral dexamphetamine [27, 52]. Intravenous dexamphetamine increases striatal dopamine concentrations in man within minutes of administration [50, 53, 54] but does not result in similarly acute changes in opioid levels in the brain [55]. A period of 1.5–3 h following dexamphetamine administration is required to reach peak endogenous opioid concentrations [15, 26, 27, 56, 57], suggesting that the mechanism of dexamphetamine-induced endogenous opioid release is also downstream of the acute dopamine release. Concentrations of other monoamines (5HT and noradrenaline) are also increased by dexamphetamine, and may play a role in endogenous opioid release in brain regions with lower dopamine transporter density, such as the frontal cortex and thalamus [15, 58]. Further work is required to better understand the mechanisms of dexamphetamine-induced endogenous opioid release, and how this is blunted in both AD and gambling disorder.

The salience of our dexamphetamine ‘reward’ may also be a factor in mediating endogenous opioid release. As addiction develops, salience towards addiction-associated cues increases, whereas responses to non-addiction related rewards decrease [59, 60]. For example, individuals with AD have blunted fMRI responses to non-salient financial rewards and higher responses to salient alcohol cues [43, 44, 61–63]. The effect of opioid antagonists to modulate the mesolimbic system also appears to be mediated by the salience of the reward. For example, naltrexone only blunts dexamphetamine-induced striatal dopamine release in rats following a period of dexamphetamine sensitisation [54]. Whereas naltrexone does not modulate striatal fMRI response to financial reward anticipation in AD [43], when the same task was performed during an alcohol infusion, a salient context, nalmefene reduces activation [11]. Naltrexone also reduces BOLD response in striatum to salient alcohol cues in alcohol-dependent individuals [10, 64]. This salience mediated endogenous opioid release, rather than low opioidergic tone, may the target for opioid receptor antagonists in reducing the risk of relapse to heavy drinking during abstinence.

Although the above evidence suggests that salience is an important factor for the activation of endogenous opioidergic signalling, a recent study has shown that feeding induces a release of endogenous opioids regardless of how palatable or ‘rewarding’ the food is [65]. One potential further study to elucidate the effect of the salience of a reward on endogenous opioid release in AD would be the administration of an alcohol challenge, where a non-blunted, or possibly enhanced, endogenous opioid release might be observed. However, this would not be an ethical experiment to conduct in our abstinent alcohol-dependent participants.

Contrary to our hypothesis we did not find higher MOR availability in alcohol-dependent participants compared with controls. Previous studies using [^11^C]carfentanil and [^11^C]diprenorphine have shown higher MOR in AD, whereas in our data there were no evidence of higher MOR availability in our alcohol-dependent participants (Fig. 2, Supplementary Table 9) [22–24]. Hermann et al. [25] reported non-significantly higher [^11^C]carfentanil binding in recently abstinent alcohol-dependent individuals and lower MOR receptor numbers in post-mortem alcohol-dependent brains measured with the MOR agonist [^3^H]DAMGO. They proposed that repeated alcohol administration in AD, and the subsequent chronic elevations in endogenous opioids, may lead to a compensatory reduction in absolute MOR numbers [25]. Chronic alcohol-induced endogenous opioid release may also lower basal endogenous opioid tone via homoeostatic feedback mechanisms, potentially through an inhibition of POMC activity [66]. This low endogenous tone, when coupled with a cessation of alcohol-induced opioid release, may lead to a relative increase in MOR availability in early abstinence, despite lower absolute MOR density.

It is unclear if there are changes in basal endogenous opioid tone or MOR receptor numbers as abstinence lengthens. Alcohol-dependent participants in our current study have a considerably longer abstinence compared with previous studies (months compared with days to weeks) [22–24] and have ‘normal’ MOR availability. This would be consistent with a ‘normalisation’ of the balance of MORs and endogenous opioid ligands as abstinence progresses. However, we did not observe any association between duration of abstinence and MOR availability in our current study and we and others have reported no changes in MOR or other opioid receptor availability in AD during the first 3 months of abstinence [22, 23]. Higher MOR availability associated with higher craving reported previously in AD may represent a greater potential to relapse, and a potential target for opioid receptor antagonist treatment. The lack of higher MOR availability in our alcohol-dependent participants may reflect their stable abstinence and low craving, and suggests these individuals may benefit less from opioid receptor antagonist treatment compared with recently abstinent individuals reporting high alcohol craving.

We explored a number of clinical variables that may influence [^11^C]carfentanil binding. Although smoking status differed in our alcohol-dependent and control groups, there was no influence of current smoking, or associated measures, on baseline MOR availability, or dexamphetamine-induced endogenous opioid release. Previous evidence concerning MOR in smokers is inconsistent, with higher, lower, or no differences in availability compared with non-smokers reported [67–69]. Consistent with others, we found that OPRM1 G-allele carriers had lower MOR availability [70, 71]. We found no significant effect of the OPRM1 polymorphism on dexamphetamine-induced endogenous opioid release, whereas others have observed lower endogenous opioid release in G-allele carriers during a pain task following placebo administration [71]. In gambling disorder, we demonstrated a positive correlation between the UPPS-P negative urgency and baseline MOR availability in the caudate [27] but we did not replicate this finding in our alcohol-dependent group. This is consistent with previous evidence showing correlations between impulsivity and [^11^C]raclopride (D2/3) and [^11^C]-( + )-PHNO (D_3_-preferring) in gambling disorder and cocaine dependence but no such correlations between [^11^C]-( + )-PHNO binding and impulsivity in AD [72–75]. This suggests that the nature of impulsivity in AD may differ from cocaine and gambling disorders.

An exploratory analysis found that higher levels of depressive symptoms (BDI score) were associated with a blunted endogenous opioid release in the amygdala, although this result does not survive a strict Bonferroni correction for multiple correlations. The amygdala is an important region for emotional processing in depression [76], and endogenous opioid release in the amygdala is associated with positive emotion and exercise-induced negative emotion, and is dysregulated in major depressive disorder [48, 77–79]. One reason for this association being observed only in alcohol-dependent participants may be due to a higher and greater range of BDI scores compared with controls. These scores were, however, low and did not reach a threshold for clinically significant depression.

Our study was designed to be adequately powered for our primary outcome of [^11^C]carfentanil ∆BP_ND_ and our samples size is consistent with those in other relevant published human PET literature examining amphetamine-induced endogenous neurotransmitter release [15, 26, 27, 50, 52, 80]. Our study sample size is too small to adequately investigate clinical factors that may be associated with our PET outcome measures.

In summary, we have demonstrated for the first time blunted dexamphetamine-induced opioid release in the brain in abstinent alcohol-dependent individuals. This study adds to the evidence supporting a role for a dysregulated opioid system in AD and builds on our previous study with the same protocol in gambling disorder showing similarly blunted dexamphetamine-induced opioid release in the presence of ‘normal’ MOR availability. The similarly blunted opioid release in gambling disorder where participants had no history of substance dependence, excluding nicotine, lends further support to an ‘opioid deficit’ contributing to vulnerability to addiction. Thus, dysregulated opioid signalling appears to be a common feature across both behavioural and substance addictions.

Further characterisation of the endogenous opioid tone and the dopamine–opioid interactions will inform our understanding of substance and behavioural addictions and how best to optimise and develop the use of opioid antagonists in treatment.

## Electronic supplementary material

Supplemental Material
