# Supplementary material for: Blunted endogenous opioid release following an oral dexamphetamine challenge in abstinent alcohol-dependent individuals
Source: Mol Psychiatry. 2018 Jun 25;25(8):1749–58. doi: 10.1038/s41380-018-0107-4 (PMC6169731; doi:10.1038/s41380-018-0107-4)
Supplement: Supplementary file 1 — Supplemental Material [file 41380_2018_107_MOESM1_ESM.docx]

*Supplementary Table 1 – Injected [^11^C]carfentanil cold mass and activity (Mean ±SD)*

|  | Healthy Controls | Alcohol dependence | *p*-value (two-tailed) |
| --- | --- | --- | --- |
| Pre-dexamphetamine injected mass | 1.33μg (±0.059) | 1.69μg (±0.47) | 0.093 |
| Post-dexamphetamine injected mass | 1.31μg (±0.58) | 1.69μg (±0.39) | 0.061 |
| Pre-dexamphetamine injected activity | 194.6mBq (±67.1) | 218.3mBq (±48.8) | 0.302 |
| Post-dexamphetamine injected activity | 197.0mBq (±65.3) | 254.1mBq (±54.3) | **0.019** |

*Supplementary Table 2 – Global Movement Parameters Mean (±SD) values for within frame cumulative movement across PET scan on X,Y,Z axis (mm) and roll, pitch and yaw rotation (degrees).*

| Movement Parameter | AD | | HV | |
| --- | --- | --- | --- | --- |
|  | Pre | Post | Pre | Post |
| X | 3.4 ±2.7 | 4.3 ±2.5 | 3.1 ±2.0 | 3.1 ±2.1 |
| Y | 11.6 ±7.5 | 11.8 ±6.9 | 11.5 ±12.3 | 12.2 ±7.2 |
| Z | 16.5 ±8.2 | 15.6 ±6.5 | 15.7 ±5.9 | 16.0 ±5.1 |
| Roll | 7.6 ±6.9 | 7.3 ±4.2 | 7.9 ±6.2 | 7.6 ±5.3 |
| Pitch | 2.2 ±1.6 | 2.6 ±1.8 | 2.4 ±1.9 | 2.0 ±1.3 |
| Yaw | 3.1 ±2.1 | 3.3 ±2.2 | 2.9 ±2.4 | 4.1 ±3.2 |

*Supplementary Table 3 – Mixed Model ANOVA examining effect of Scan (pre- or post-dexamphetamine and Status (AD or HC) on Global Movement Parameters on X,Y,Z axis movement and roll, pitch and yaw rotation.*

|  |  | F-ratio (effect *df*, error *df*) | p value |
| --- | --- | --- | --- |
| **X, Y, Z model** |  |  |  |
| Within-subject effects | *Scan* | 0.10 (1, 26) | 0.755 |
|  | *Scan x Status* | 0.01 (1, 26) | 0.924 |
|  | *Movement* | 17.9 (1.3, 33.0) | **<0.001** |
|  | *Movement x Status* | 0.06 (1.3, 33.0) | 0.866 |
|  | *Scan x Movement* | 0.61 (1.4, 36.0) | 0.493 |
|  | *Scan x Movement x Status* | 0.43 (1.4, 36.0) | 0.579 |
| Between-subject effects | *Status* | 0.04 (1, 26) | 0.853 |
|  |  |  |  |
| **Roll, Pitch, Yaw model** |  |  |  |
| Within-subject effects | *Scan* | 0.06 (1, 26) | 0.802 |
|  | *Scan x Status* | 0.03 (1, 26) | 0.870 |
|  | *Movement* | 38.5 (1.8, 46.6) | **<0.001** |
|  | *Movement x Status* | 0.04 (1.8, 46.6) | 0.945 |
|  | *Scan x Movement* | 0.14 (1.3, 33.3) | 0.775 |
|  | *Scan x Movement x Status* | 0.27 (1.3, 33.3) | 0.664 |
| Between-subject effects | *Status* | 0.03 (1, 26) | 0.875 |

*Supplementary Figure 4* – Plasma dexamphetamine concentrations (ng/ml) in HC and AD (mean ± SD)

*Supplementary Table 5 – Serum Cortisol and Plasma amphetamine concentrations mixed model ANOVA*

|  |  | F-ratio (effect *df*, error *df*) | p value |
| --- | --- | --- | --- |
| **Plasma Dexamphetamine** |  |  |  |
| Within-subject effects | *Time* | 100.492 (2.6, 57.0) | **<0.001** |
|  | *Time x Status* | 1.323 (2.6, 57.0) | 0.276 |
| Between-subject effects | *Status* | 4.047 (1, 22) | 0.057 |
|  |  |  |  |
| **Serum Cortisol** |  |  |  |
| Within-subject effects | *Time* | 25.115 (2.4, 36.3) | **0.001** |
|  | *Time x Status* | 0.381 (2.4, 36.3) | 0.725 |
| Between-subject effects | *Status* | 1.602 (1, 15) | 0.225 |

*Supplementary Figure 6* – Dexamphetamine induced cortisol release in HC and AD (mean ± SD)

*Supplementary Table 7 – SAIRS mixed model ANOVA*

|  |  | F-ratio (effect *df*, error *df*) | p value |
| --- | --- | --- | --- |
| **Euphoria** |  |  |  |
| Within-subject effects | *Time* | 2.510 (3.0, 79.1) | 0.064 |
|  | *Time x Status* | 0.584 (3.0, 79.1) | 0.630 |
| Between-subject effects | *Status* | 1.154 (1, 26) | 0.293 |
|  |  |  |  |
| **Alert** |  |  |  |
| Within-subject effects | *Time* | 1.589 (2.8, 73.2) | 0.202 |
|  | *Time x Status* | 0.346 (2.8, 73.2) | 0.780 |
| Between-subject effects | *Status* | 0.381 (1, 26) | 0.543 |
|  |  |  |  |
| **Restless** |  |  |  |
| Within-subject effects | *Time* | 0.809 (2.6, 66.4) | 0.476 |
|  | *Time x Status* | 1.408 (2.6, 66.4) | 0.251 |
| Between-subject effects | *Status* | 2.838 (1, 26) | 0.104 |
|  |  |  |  |
| **Anxiety** |  |  |  |
| Within-subject effects | *Time* | 2.230 (3.0, 78.2) | 0.091 |
|  | *Time x Status* | 3.862 (3.0, 78.2) | **0.012** |
| Between-subject effects | *Status* | 3.788 (1, 26) | 0.062 |
|  |  |  |  |

*Supplementary Table 8 – Results from mixed model ANOVAs examining [^11^C]carfentanil BP_ND_ and ∆BP_ND_ in AD and HC participants*

|  |  | F-ratio (effect *df*, error *df*) | p value |
| --- | --- | --- | --- |
| **Pre- and post-dexamphetamine model in AD and HC** |  |  |  |
| Within-subject effects | *Scan* | 13.145 (1, 26) | **0.001** |
|  | *Scan x Status* | 17.077 (1, 26) | **<0.001** |
|  | *ROI* | 212.457 (3.9, 102.2) | **<0.001** |
|  | *ROI x Status* | 0.455 (3.9, 102.2) | 0.765 |
|  | *Scan x ROI* | 1.839 (3.7, 94.9) | 0.134 |
|  | *Scan x ROI x Status* | 1.382 (3.7, 94.9) | 0.249 |
|  |  |  |  |
| **Baseline BP_ND_ model in AD and HC** |  |  |  |
| Within-subject effects | *ROI* | 224.1 (4.4, 114.7) | **<0.001** |
|  | *ROI x Status* | 0.700 (4.4, 114.7) | 0.709 |
| Between-subject effects | *Status* | 2.336 (1, 26) | 0.139 |
|  |  |  |  |
| **[^11^C]carfentanil ∆BP_ND_ model in AD and HC** |  |  |  |
| Within-subject effects | *ROI* | 2.054 (3.5, 91.1) | 0.102 |
|  | *ROI x Status* | 0.401 (3.5, 91.1) | 0.782 |
| Between-subject effects | *Status* | 14.328 (1, 26) | **0.001** |
|  |  |  |  |
| **[^11^C]carfentanil ∆BP_ND_ model in AD and HC with smoking status as covariate** |  |  |  |
| Within-subject effects | *ROI* | 1.581 (3.596, 86.311) | 0.192 |
|  | *ROI x Status* | 0.106 (3.596, 86.311) | 0.973 |
|  | *ROI x Smoking* | 0.311 (3.596, 86.311) | 0.851 |
| Between-subject effects | *Status* | 11.067 (1,24) | **0.003** |
|  | *Smoking* | 0.159 (1,24) | 0.693 |

*Supplementary Table 9 – Pre- and post-dexamphetamine [^11^C]carfentanil BP_ND_ and [^11^C]carfentanil ∆BP_ND_ values (mean ±SD) in abstinent alcohol dependent and healthy control participants including Effect size calculations (Cohen’s D) for significant ROI* ∆BP_ND_ *differences between AD and HC.*

| Region of interest | Pre-dexamphetamine  [^11^C]carfentanil BP_ND_ | | Post-dexampheramine  [^11^C]carfentanil BP_ND_ | | [^11^C]carfentanil ∆BP_ND_ | | |
| --- | --- | --- | --- | --- | --- | --- | --- |
|  | AD | HC | AD | HC | AD | HC | Effect Size (Cohen’s D) |
| Cerebellum | 0.79  (±0.17) | 0.83††  (±0.31) | 0.80  (±0.16) | 0.78††  (±0.29) | 0.016*  (±0.089) | -0.052*  (±0.049) | 0.94 |
| Frontal Lobe | 1.03  (±0.09) | 1.12††  (±0.16) | 1.05  (±0.12) | 1.06††  (±0.15) | 0.017**  (±0.062) | -0.052**  (±0.033) | 1.36 |
| Insula | 1.43  (±0.13) | 1.49††  (±0.20) | 1.46  (±0.18) | 1.41††  (±0.20) | 0.021**  (±0.070) | -0.049**  (±0.034) | 1.28 |
| Thalamus | 1.58  (±0.17) | 1.68††  (±0.23) | 1.59  (±0.17) | 1.58†† (±0.22) | 0.012**  (±0.071) | -0.052**  (±0.028) | 1.19 |
| Amygdala | 1.57  (±0.19) | 1.72†  (±0.25) | 1.56  (±0.18) | 1.62†  (±0.19) | -0.004  (±0.078) | -0.047  (±0.059) | 0.62 |
| Anterior Cingulate | 1.37  (±0.11) | 1.48††  (±0.19) | 1.40  (±0.18) | 1.41††  (±0.18) | 0.020**  (±0.062) | -0.046**  (±0.027) | 1.39 |
| Caudate | 1.30  (±0.26) | 1.37††  (±0.37) | 1.27  (±0.29) | 1.27††  (±0.36) | -0.029  (±0.077) | -0.073  (±0.066) | 0.61 |
| NAcc | 2.64  (±0.31) | 2.84†† (±0.39) | 2.66  (±0.37) | 2.67††  (±0.35) | 0.007*  (±0.064) | -0.059*  (±0.051) | 1.13 |
| Putamen | 1.83  (±0.16) | 1.88††  (±0.27) | 1.82  (±0.18) | 1.77†† (±0.30) | -0.003**  (±0.054) | -0.060**  (±0.037) | 1.23 |
| Hypothalamus | 1.40  (±0.36) | 1.58†  (±0.35) | 1.39  (±0.39) | 1.47†  (±0.44) | -0.008  (±0.102) | -0.089  (±0.107) | 0.77 |

*AD vs. HC *p<0.05, **p<0.005*

*HC pre-dexamphetamine vs. HC post-dexamphetamine* †*p<0.05,* ††*p<0.005*

*Supplementary Figure 10*

***A*** – Effect of OPRM1 polymorphism on baseline [^11^C]carfentanil BP_ND_ in whole study population (HC & AD) (A:A n=18, G:A/G:G n=6) (*differences in BP_ND_ between G allele and non-G allele carriers *p*<0.005, mean ± SD)


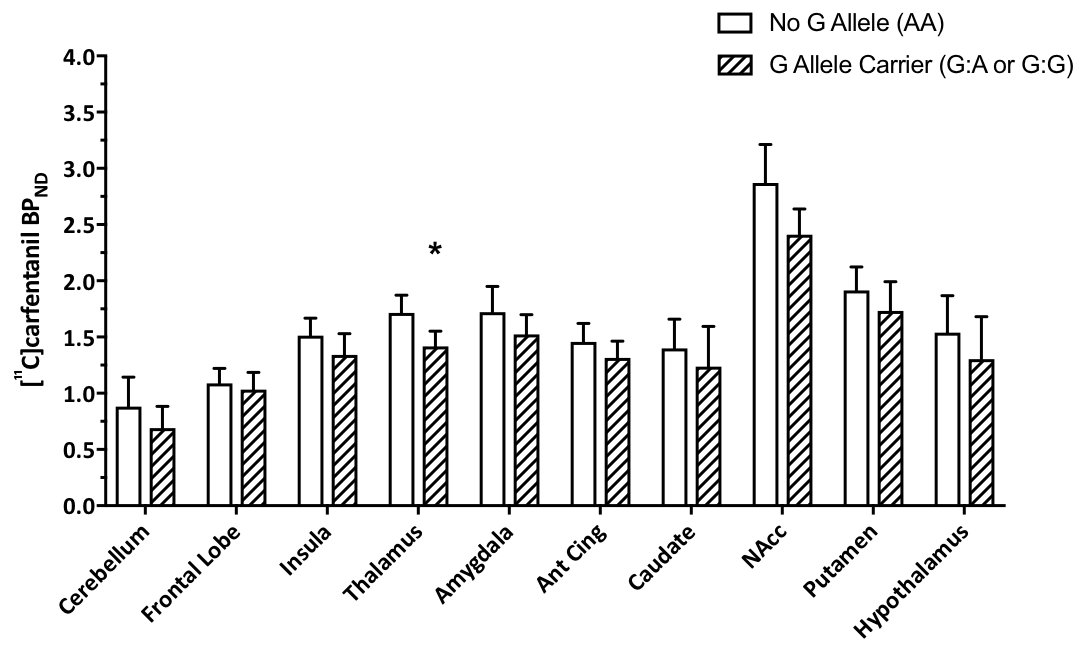


***B*** – Effect of OPRM1 polymorphism on [^11^C]carfentanil ∆BP_ND_ in whole study population (HC & AD) (A:A n=18, G:A/G:G n=6, mean ± SD)


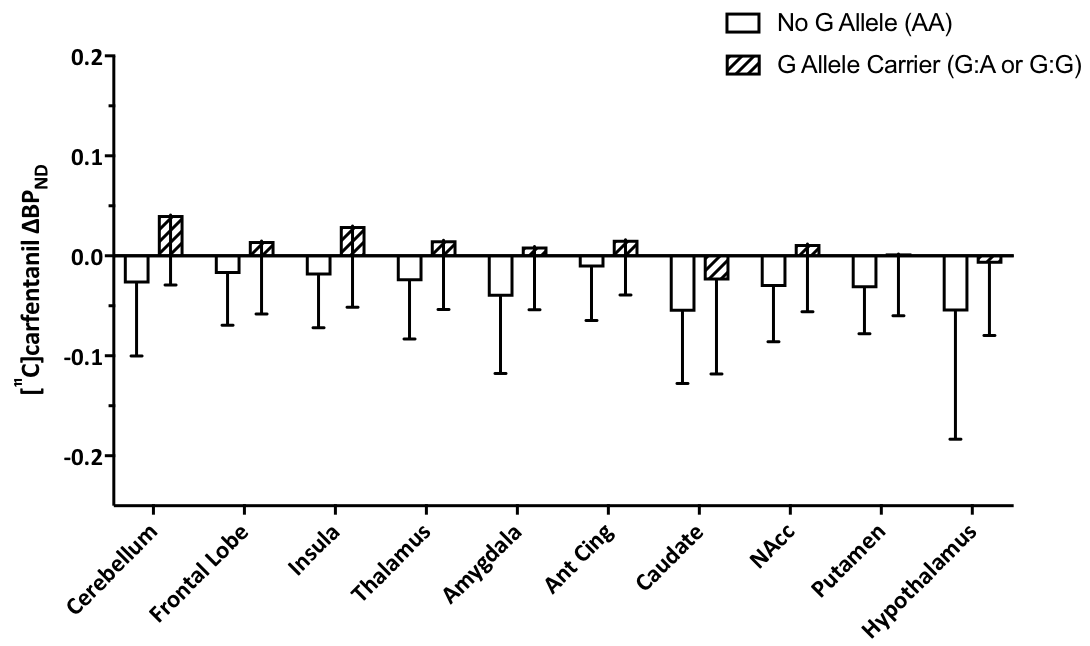


*Supplementary Table 11 – OPRM1 polymorphism mixed model ANOVA*

|  |  | F-ratio (effect *df*, error *df*) | p value |
| --- | --- | --- | --- |
| **Genotype and Baseline BP_ND_ model in AD and HC** |  |  |  |
| Within-subject effects | *ROI x Genotype* | 1.573 (3.9, 78.1) | 0.191 |
|  | *ROI x Status x Genotype* | 0.921 (3.9, 78.1) | 0.454 |
| Between-subject effects | *Genotype* | 5.651 (1, 20) | **0.028** |
|  | *Status x Genotype* | 0.018 (1, 20) | 0.894 |
|  |  |  |  |
| **Genotype and ∆BP_ND_ model in AD and HC** |  |  |  |
| Within-subject effects | *ROI x Genotype* | 0.266 (2.9, 58.5) | 0.845 |
|  | *ROI x Status x Genotype* | 0.693 (2.9, 58.5) | 0.557 |
| Between-subject effects | *Genotype* | 1.791 (1, 20) | 0.196 |
|  | *Status x Genotype* | 0.472 (1,20) | 0.500 |

*Supplementary Table 12 – [^11^C]carfentanil BP_ND_ values (mean ±SD) in individuals with A:A or A:G/G:G OPRM1 polymorphism and independent sample t-test p-value (two-tailed Bonferroni corrected p<0.005)*

| ROI | Genotype | | *p* value |
| --- | --- | --- | --- |
|  | A:A | A:G or G:G |  |
| Cerebellum | 0.88  (±0.25) | 0.69  (±0.18) | 0.114 |
| Frontal Lobe | 1.09  (±0.13) | 1.03  (±0.14) | 0.411 |
| Insula | 1.51  (±0.15) | 1.34  (±0.17) | 0.036 |
| Thalamus | 1.71  (±0.15) | 1.42  (±0.12) | <0.001 |
| Amygdala | 1.72  (±0.22) | 1.52  (±0.16) | 0.068 |
| Anterior  Cingulate | 1.46  (±0.16) | 1.32  (±0.13) | 0.076 |
| Caudate | 1.40  (±0.25) | 1.24  (±0.33) | 0.237 |
| Nucleus Accumbens | 2.87  (±0.33) | 2.41  (±0.21) | 0.006 |
| Putamen | 1.91  (±0.20) | 1.73  (±0.24) | 0.094 |
| Hypothalamus | 1.54  (±0.32) | 1.30  (±0.34) | 0.157 |

**ARCHITECT serum Cortisol assay**

This is a chemiluminescent microparticle immunoassay (CMIA) for the quantitative determination of cortisol in human serum, plasma or urine on the ARCHITECT iSystem.

The ARCHITECT Cortisol assay is designed to have an assay precision of ≤ 10% total CV for serum samples ≥ 3 to ≤ 35 μg/dL.

<http://www.ilexmedical.com/files/PDF/Cortisol_ARC.pdf>

**Dexamphetamine assay details**

Alongside human plasma, quality control samples (spiked plasma at 40 mg/L ) where analysed as part of each analytical sequence. Based on the results of the QC samples run throughout the duration study, the mean intra-assay coefficient of variance was 7.5%, the inter-assay coefficient of variance was 8.6%. The mean accuracy was 105%
